# Endocrine, gender dysphoria, and sexual function benefits of gender-affirming bilateral orchiectomy: patient outcomes and surgical technique

**DOI:** 10.1093/sexmed/qfae048

**Published:** 2024-08-29

**Authors:** Jenna Stelmar, Robert Victor, Nance Yuan, Shannon M Smith, Samhita Mallavarapu, Sandeep Sandhu, Maurice M Garcia

**Affiliations:** Cedars-Sinai Transgender Surgery & Health Program, Los Angeles, CA 90211, United States; Department of Urology, Cedars-Sinai Medical Center, Los Angeles, CA 90048, United States; UC San Diego School of Medicine, La Jolla, CA 92037, United States; Cedars-Sinai Transgender Surgery & Health Program, Los Angeles, CA 90211, United States; Department of Urology, Cedars-Sinai Medical Center, Los Angeles, CA 90048, United States; Department of Anesthesiology, Perioperative and Pain Medicine, Stanford University, Stanford, CA 94305, United States; Cedars-Sinai Transgender Surgery & Health Program, Los Angeles, CA 90211, United States; Department of Urology, Cedars-Sinai Medical Center, Los Angeles, CA 90048, United States; Cedars-Sinai Transgender Surgery & Health Program, Los Angeles, CA 90211, United States; Department of Urology, Cedars-Sinai Medical Center, Los Angeles, CA 90048, United States; Cedars-Sinai Transgender Surgery & Health Program, Los Angeles, CA 90211, United States; Department of Urology, Cedars-Sinai Medical Center, Los Angeles, CA 90048, United States; Cedars-Sinai Transgender Surgery & Health Program, Los Angeles, CA 90211, United States; Department of Urology, Cedars-Sinai Medical Center, Los Angeles, CA 90048, United States; Cedars-Sinai Transgender Surgery & Health Program, Los Angeles, CA 90211, United States; Department of Urology, Cedars-Sinai Medical Center, Los Angeles, CA 90048, United States

**Keywords:** gender-affirming hormone therapy, gender-affirming bilateral orchiectomy, patient satisfaction, decision-making, transgender health, genital gender-affirming surgery, gender-affirming vaginoplasty

## Abstract

**Background:**

Gender-affirming bilateral orchiectomy (GABO) may be completed as either a standalone procedure (sGABO) or at the same time as gender-affirming vaginoplasty (vGABO). GABO is postulated to decrease gender-affirming hormone therapy (GAHT) dosages and reduce gender dysphoria, but these phenomena are not empirically described in the medical literature.

**Aim:**

The primary aim of this study was to describe changes in GAHT dosages after sGABO and vGABO. A secondary aim was to assess sGABO patients’ preoperative decision-making priorities and postoperative satisfaction.

**Methods:**

A retrospective chart review identified 204 patients who completed GABO as either a standalone procedure (64% of patients) or at the same time as vaginoplasty (36%). Patient demographic data, surgical outcomes, and pre- and postoperative GAHT dosage data were recorded. Patients completed an opinion questionnaire to assessed decision-making priorities, as well as postoperative satisfaction and changes in quality-of-life measures.

**Outcomes:**

Primary outcomes included pre- and postoperative dosages of estradiol, progesterone, and spironolactone. Secondary outcomes included sGABO patient priorities, satisfaction with sGABO, changes in quality-of-life measures between sGABO and vGABO patients, and sGABO recommendations to future patients.

**Results:**

The sGABO and vGABO patients experienced a statistically significant dosage reduction in all three GAHT assessed: estradiol, progesterone, and spironolactone (*P* < .05). All patients discontinued spironolactone postoperatively. Zero complications related to GABO were recorded for patients in either group. The patient questionnaire revealed that sGABO patients prioritize decreasing endogenous testosterone and reducing their GAHT as most important in their decision to undergo sGABO prior to vaginoplasty. A majority of sGABO patients reported improvement in all nine quality-of-life indices. None of the sGABO patients would recommend against sGABO to a friend who is waiting for vaginoplasty.

**Clinical Implications:**

For patients who are interested in vaginoplasty, sGABO may serve as a more immediate, low-risk, intermediary step that comes with the benefits of GABO, including significant GAHT medication reduction and gender dysphoria relief.

**Strengths and Limitations:**

This study offers a comprehensive evaluation of the impact of GABO on patients, combining empirical data with subjective patient feedback. Limitations include the retrospective design and the use of unvalidated survey questions.

**Conclusion:**

Prevaginoplasty GABO is a viable option to more immediately alleviate gender dysphoria and reduce GAHT medications for patients who are interested in gender-affirming vaginoplasty.

## Introduction

Patients assigned male sex at birth who desire genital gender-affirming surgery (gGAS) have different options to choose from. They may elect to undergo gender-affirming bilateral orchiectomy (GABO) with or without further vaginoplasty, which can be completed with or without creation of a vaginal canal.^1–6^ While GABO is typically performed at the same time as vaginoplasty (vGABO), it can also be completed at any time point before vaginoplasty as a standalone procedure (sGABO).^7^^,^^8^ The operative time for sGABO is approximately 30 minutes and it is typically offered as an outpatient surgery, with patients returning home on the same day of surgery. In contrast, vGABO surgery typically takes 3-6 hours and requires a 3-7 day inpatient stay for postoperative monitoring and bedrest (times vary depending on whether a vaginal canal is created).^9–11^ vGABO is associated with the longest wait times of any feminizing gender-affirming surgery, with high-volume centers reporting 1-3–year waitlists due to the steadily increasing number of patients seeking gender-affirming vaginoplasty.^12–15^ Vaginoplasty furthermore requires preoperative permanent hair removal from the penis shaft and scrotum, which takes a mean ± SD time of 9.6 ± 6.7 months with electrolysis, and 10.2 ± 3.3 months with laser hair removal.^16^

GABO removes the native testes, reducing patient gender dysphoria and minimizing endogenous testosterone production.^17^ It has been posited that patients can reduce the dosages of their estradiol, androgen blockers, and progesterone gender-affirming hormone therapy (GAHT) regimens after GABO, but this phenomenon has not been described or quantified in the literature.^8^ Given that side effects of GAHT are proportional to the dose administered, GABO may offer a significant benefit in minimizing adverse effects.^18^^,^^19^ Estradiol, in particular, has been linked to an increased risk of cardiovascular events, including deep vein thrombosis, myocardial infarction, pulmonary embolism, and hypertension.^20^^,^^21^ Progesterone, while not recommended in the World Professional Association for Transgender Health Standards of Care Version 8 and only used by a subset of patients, may have the undesired effects of drowsiness, nausea, weight gain, depression, and lipid changes, with an increased risk of thromboembolism.^1^^,^^22^^,^^23^ Androgen blockers, such as spironolactone, may also come with the added side effects of polyuria, polydipsia, postural hypotension, and hyperkalemia, especially in patients with impaired renal function or those taking other potassium-retaining drugs, like angiotensin-converting enzyme inhibitors.^24^

Patients interested in vaginoplasty must decide whether to complete GABO as an earlier standalone procedure or at the same time as vaginoplasty. This decision-making process requires weighing the *upside* of a more immediate relief from gender dysphoria and the benefits associated with reduced GAHT dosages against the *downside* of undergoing an additional outpatient surgical procedure.^7^^,^^8^^,^^17^ There is a gap in the medical literature that describes both the preoperative goals and postoperative satisfaction of sGABO patients, especially in comparison to vGABO patients. This information could provide valuable first-person insight into the lived experiences and outcome reflections of patients who have navigated a similar decision.

This study we explores the following two hypotheses: (1) Do both sGABO and vGABO patients experience a statistically significant reduction in all three GAHTs (estradiol, progesterone, and spironolactone)? (2) Will sGABO and vGABO patients report similar postoperative satisfaction across multiple gender dysphoria indices? The primary aim of the present study was to assess the potential benefits of sGABO by quantifying and comparing changes in estradiol, progesterone, and spironolactone dosages that occur after sGABO and vGABO. A secondary aim was to describe sGABO patients’ preoperative expectations, goals, and postoperative satisfaction, and to compare their self-reported postoperative satisfaction measures to those of vGABO patients.

## Methods

All consecutive patients assigned male sex at birth who underwent gGAS at a single academic center between March 2017 and September 2023 were identified. All patients met current World Professional Association for Transgender Health criteria (Standards of Care Version 7/8).^1^ These include significant, sustained gender incongruence with a diagnosis of gender dysphoria; a minimum of 6 months of GAHT (unless it is not desired and/or medically contraindicated); two letters of support; the capacity to understand and consent to the risks and benefits of sGABO versus vGABO; an understanding of the irreversible impact of GABO on reproduction and exploration of reproductive options if desired; and well-controlled medical/mental health conditions.

### Gender-affirming bilateral orchiectomy surgical technique

All GABOs were performed using the technique described below and in Video 1:

1. Mark the midline of the scrotum and use a scalpel to make a single 2.5-cm vertical incision (skin only) over the median raphe of the anterior scrotum. Then push the testicle up to the surface so that the layer of dartos fascia is taut.
2. With the Bovie instrument on cut (35), incise through all tissue layers up to the surface of the testicle (e.g., visceral layer of tunica vaginalis).
3. Place a Kelly clamp between the now opened edge of the parietal tunica vaginalis. Push the clamp tip through the closed processus vaginalis while keeping it parallel to the cord, then spread the clamp. Use Bovie cautery to incise the tissues at the 12 o'clock position on the cord, proximally, to the base of the stretched cord.
4. Apply Bovie cautery to incise the remaining parietal tunica vaginalis attachments to the testicle.
5. Use a finger covered by knitted gauze to gently sweep all tissue layers superficial to the internal spermatic fascia proximally toward the base of the stretched cord. This leaves the cord skeletonized and covered by only internal spermatic fascia. Note that the sensory branches of the ilioinguinal nerve and genitofemoral nerve and the sympathetic nerve branches of the testicular plexus all travel within cord tissues superficial to the internal spermatic fascia at the 3, 6, and 9 o'clock positions. These sensory nerve branches are preserved by incising the cord at 12 o'clock and sweeping these intact tissues proximally toward the base of the cord*.*
6. Place a Pean clamp across the stretched spermatic cord, approximately 1 inch distal to the external inguinal ring.
7. Inject 10 mL of 0.5% bupivacaine into the spermatic cord *proximal* to the Pean clamp to provide a regional sensory nerve block. Release traction on the cord while injecting so that the stump can accommodate more anesthetic.
8. Suture-ligate the cord with two separate circumferential sutures (2-0 Vicryl), placed in series proximal to the Pean clamp. Place the first suture proximally, furthest from the clamp. Then place the second suture adjacent to the clamp. Briefly release (ie, “flash”) the Pean clamp as the circumferential is drawn through the second suture as tightly as possible. Leave a long tail of the second suture intact to serve as a “leash” to maintain connection to the stump.
9. After confirming complete hemostasis within the field, place a second Pean clamp on the cord 1 cm distal to the first clamp.
10. Transect the cord between the clamps using a 15-blade scalpel, taking care to leave 3-4 mm of cord tissue on the proximal clamp. Pass the testicle and cord (still secured with the distal Pean clamp) off the field.
11. Next, use Bovie cautery to fulgurate the structures within the cut end of cord tissue left that is exposed on the surface of the proximal clamp.
12. Repeat inspection for bleeding in the field. If all bleeding has been controlled, release the Pean clamp and reinspect for any new bleeding.
13. Once complete hemostasis is confirmed, irrigate the wound with antibiotic-infused saline.
14. Remove the contralateral testicle through the same skin incision by repeating the steps above.
15. Finally, close the wound in two layers. For the deep layer, place two figure-of-eight 5-0 Vicryl sutures to loosely approximate adipose and dartos tissues and subcutaneous tissues (for hemostasis within these tissue layers). Reapproximate the skin layer using a single, running locking 5-0 Vicryl suture.
16. Wash and dry the incision site and then dress it with surgical glue. Unroll and unfold two Kerlix knitted gauze rolls to yield a large gauze fluff and apply them to the wound area. Apply direct pressure to the scrotum by placing athletic support strap to help prevent hematoma formation.
17. Patients are typically discharged home on the same day as the surgery.

### Retrospective chart review of changes in GAHT

In addition to relevant demographic and clinical characteristics, pre- and postoperative GAHT medication dosages and routes of intake were documented during the retrospective chart review. The GAHT medications included estradiol, progesterone, and spironolactone. All GAHT management was performed independently by GAHT care providers unrelated to the authors and this study. The Cedars-Sinai Institutional Review Board approved this study (PRO #0005933, Study #00001611). Patient consent was indicated by completion of the anonymous online questionnaire.

### Qualitative patient questionnaire

The patient questionnaire was administered during the earlier study months of August and September 2021. All 170 patients who had been identified in the GAHT chart review by this time were invited to complete an anonymous, online questionnaire via Qualtrics (Provo, UT, United States). The questionnaire consisted of 24 questions that assessed all respondents’ gGAS history and future plans for gGAS, as well as their postoperative satisfaction in the context of gender dysphoria improvements (Supplemental Material 1). Patients who underwent sGABO rated and ranked the importance of various preoperative expectations/goals that influenced their choice of sGABO over vGABO, and their satisfaction with completing sGABO.

### Statistical analysis

Data from the retrospective chart review were analyzed using IBM SPSS Statistics v24 (Chicago, IL, United States). Fisher’s exact tests and *t*-tests were used to assess for between-group differences in demographic characteristics. Wilcoxon signed-rank tests were used to assess for pre- versus postoperative GAHT dosage changes within subjects. To limit confounding, patients were excluded from statistical analyses if they had incomplete dosage data, changed the route of administration (e.g., from oral to intramuscular [IM]) and/or were taking a formulation or route of administration with a limited sample size. Results from the patient questionnaire were analyzed using Qualtrics Stats iQ software. Differences between sGABO and vGABO patients were defined as statistically significant at *P* < .05 using a Student *t*-test with a 2-tailed alpha = .05 and power = 0.80.

## Results

### Retrospective chart review

#### Demographic and clinical characteristics

A total of 204 patients were identified: 130/204 (64%) completed sGABO and 74/204 (36%) vGABO. There was no statistically significant difference in age between groups (sGABO: 36.6 ± 13.5 and vGABO 39.5 ± 14.7 years, *P* > .05). The sGABO patients had a significantly higher mean body mass index at the time of surgery (27.0 ± 6.4 vs 25.2 ± 4.7, *P* = .02) and were significantly more likely to identify as nonbinary (6% nonbinary vs 0% nonbinary, *P* < .05) **(**Table 1**).**

**Table 1:** Gender-affirming bilateral orchiectomy patient characteristics.^a^

		**sGABO Patients** **(n = 130)**	**vGABO Patients** **(n = 74)**	***P*-value**
Age, mean ± SD		36.6 ± 13.5 y	39.5 ± 14.7 y	.20
BMI, mean ± SD		27.0 ± 6.4	25.2 ± 4.7	.02^*^
Gender identity				<.05^*^
	Transgender woman	94%	100%	
	Nonbinary	6%	0%	
Smoking history				.80
	Never	79%	78%	
	Former	10%	7%	
	Current	11%	15%	

Abbreviations: BMI, body mass index; sGABO, gender-affirming bilateral orchiectomy completed as a standalone procedure; vGABO, gender-affirming bilateral orchiectomy completed at the same time as vaginoplasty.

^a^Demographic and clinical characteristics of n = 204 patients who completed sGABO (n = 130) and vGABO (n = 74) at a single academic institution.

#### Pre- versus postoperative GAHT doses

##### Estradiol

Of the 130 sGABO patients, 27 were excluded from further analyses (16 changed route of administration, 7 had incomplete data, 2 were taking IM estradiol cypionate, and 2 transdermal estradiol). The remaining 103 sGABO patients took oral estradiol (n = 64, 62%) or IM estradiol valerate (n = 39, 38%).

Of the 74 vGABO patients, 29 were excluded (11 changed route of administration, 10 had incomplete data, 4 were taking IM estradiol cypionate, and 4 transdermal estradiol). The remaining 45 vGABO patients took oral estradiol (n = 31, 69%) or IM estradiol valerate (n = 14, 31%). 

**Figure 1:**
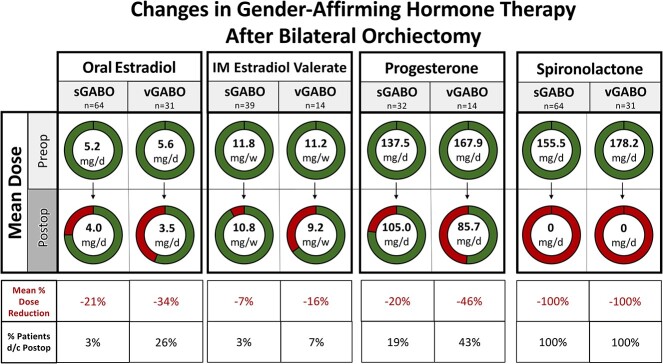
Gender-affirming bilateral orchiectomy completed as a standalone procedure (sGABO) and at the same time as vaginoplasty (vGABO) both resulted in significant reduction of baseline estrogen, spironolactone, and progesterone dosages (all *P* < .05).

Patients in both sGABO and vGABO groups experienced a statistically significant reduction in their pre- versus postoperative estradiol doses (both *P* < .05), for both oral and IM valerate routes of administration (Figure 1). Due to limited sample size, patients taking other formulations and/or routes of estradiol administration were not included in dose reduction analyses.

#### Progesterone

Progesterone was taken preoperatively by 32/103 (31%) sGABO and 14/45 (31%) vGABO patients. There was a statistically significant reduction in postoperative progesterone in both groups (both *P* < .02).

#### Spironolactone

Spironolactone was taken by 64/103 (62%) sGABO patients and 31/45 (69%) vGABO patients. There was a statistically significant reduction in postoperative spironolactone in both groups (both *P* < .01).

#### Complications

No intraoperative or perioperative complications were recorded for sGABO patients that required medical, surgical, or activity-restricting interventions. In the vGABO group, zero (0%) patients had complications related to the bilateral orchiectomy portion of their surgery (e.g., delayed bleeding from the transected spermatic cord-stump). Zero (0%) sGABO patients expressed any concerns regarding the aesthetics of their healed orchiectomy scar at follow up.

### Anonymous patient questionnaire

A total of 136/170 patients completed the study questionnaire (response rate = 80%). Fifty of 136 (37%) respondents had undergone vGABO and 86/136 (63%) sGABO. Of the sGABO respondents, 34/86 (40%) reported they had already completed a later gender-affirming vaginoplasty, 35/86 (41%) planned to undergo vaginoplasty in the future, 8/86 (9%) had no interest in vaginoplasty, and 9/86 (10%) were undecided. The median reported time interval between sGABO and subsequent vaginoplasty was 12 months (range: 6-120 months).

#### sGABO patients’ ranked and rated preoperative expectations

**Figure 2:**
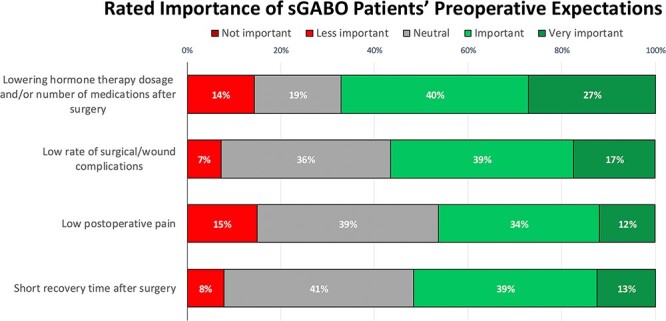
Standalone gender-affirming bilateral orchiectomy (sGABO) patients reported variability in the rated importance of five preoperative expectations/goals; however, decreasing GAHT after surgery was rated important/very important by the most respondents.

sGABO patients rated the potential to lower or discontinue GAHT as most important in their decision to complete an earlier orchiectomy (Figure 2). Similarly, the highest ranked preoperative motivating factor for patients was elimination of their endogenous testosterone and its various masculinizing effects.

#### Comparing sGABO versus vGABO patients’ postoperative changes in body image

**Figure 3:**
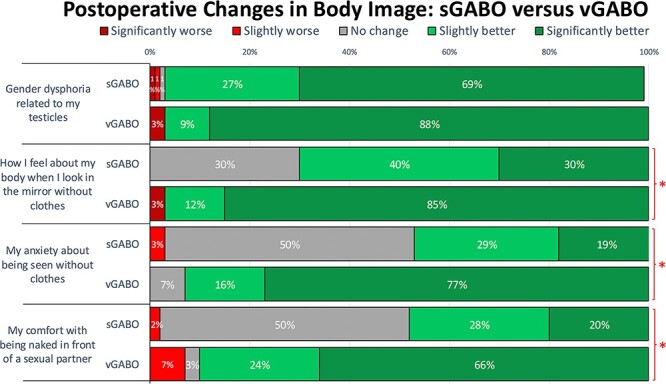
There was a statistically significant difference between respondents who completed gender-affirming bilateral orchiectomy as a standalone procedure (sGABO) and at the same time as vaginoplasty (vGABO) in 3 of 4 body image domains after surgery (^*^ = *P* < .05).

vGABO patients reported significantly greater postoperative improvement in 3/4 body image domains, compared to sGABO patients (all *P* < .01) (Figure 3). Over 96% of respondents in each group reported reduced gender dysphoria (*P* > .05).

#### Comparing postoperative changes in GAHT experience

**Figure 4:**
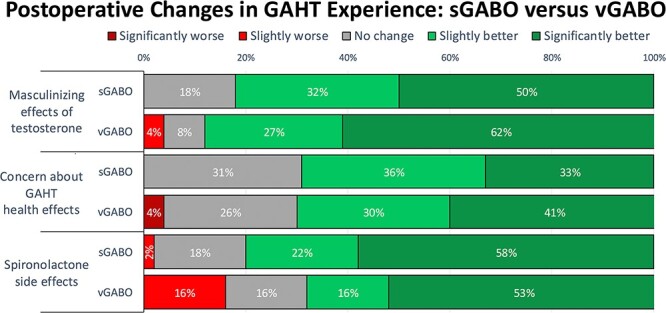
There was no significant difference in any of the 3 assessments of postoperative experience with gender-affirming hormone therapy between patients who completed gender-affirming bilateral orchiectomy as a standalone procedure (sGABO) versus at the same time as vaginoplasty (vGABO).

There was no statistically significant between-group difference in any of the three domains related to postoperative experience with GAHT (Figure 4).

#### Comparing postoperative changes in mental health

**Figure 5:**
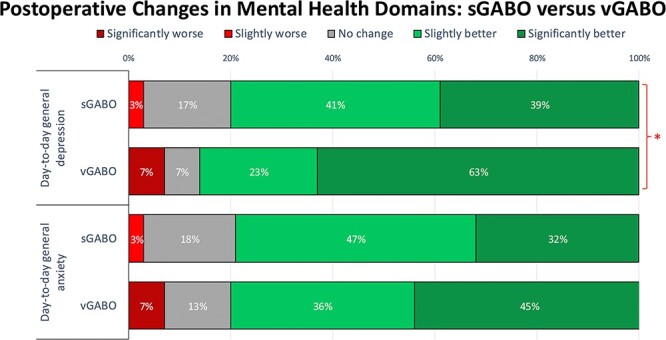
Self-reported improvement in day-to-day depression was significantly higher among patients who completed gender-affirming bilateral orchiectomy at the same time as vaginoplasty (vGABO) as compared to as a standalone procedure (sGABO) (^*^ = P < .05). However, the proportion of patients that reported any improvement was similar among both groups (80% vs 86%, respectively). Improvement in day-to-day levels of anxiety was high among both sGABO and vGABO groups (79% vs 81%; *P* > .05).

Over 80% of respondents in both groups reported improvements in their day-to-day general depression and anxiety (Figure 5). vGABO respondents reported greater improvement in their day-to-day general depression (*P* < .05).

#### sGABO patient satisfaction with pain control, appearance, and time to recovery

**Figure 6:**
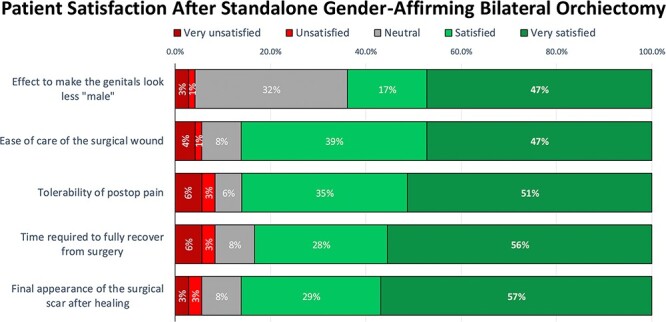
A majority of patients who underwent standalone gender-affirming bilateral orchiectomy (sGABO) reported satisfaction across five different domains related to postoperative appearance, wound care, pain control, recovery time, and surgical site cosmesis.

A majority of sGABO patients reported high postoperative satisfaction across the five measured domains of appearance, pain control, and recovery time (Figure 6).

#### sGABO patient attitudes toward pre-vaginoplasty orchiectomy

When asked, “Would you recommend a pre-vaginoplasty orchiectomy to a friend who is considering vaginoplasty?” 76% of sGABO respondents confirmed that they would recommend sGABO and 24% responded “maybe.” A follow-up question found that 93% of sGABO patients who completed later vaginoplasty reported that a pre-vaginoplasty orchiectomy was “worth it.”

Patients were also asked, “Up to how many months before vaginoplasty surgery would you say it is still' ‘worth it’ to undergo pre-vaginoplasty bilateral orchiectomy?*”* The responses were as follows: 4% reported ≥1 month; 4% ≥3 months; 4% ≥4 months; 22% ≥6 months; 8% ≥8 months; 4% ≥10 months; 4% ≥11 months; and 50% ≥12 months.

## Discussion

This study describes changes in gender-affirming hormone therapy dosages after bilateral orchiectomy completed as either a standalone procedure or concurrently with vaginoplasty, as well as patients’ preoperative expectations and postoperative changes in quality of life and satisfaction. In support of our hypotheses, a statistically significant dose reduction in both groups was observed in estradiol, progesterone, and spironolactone, with all patients discontinuing spironolactone after surgery. A majority of sGABO patients reported improvement in all nine indices of gender dysphoria and quality of life. Zero sGABO patients would recommend against their choice of an earlier pre-vaginoplasty orchiectomy.

Findings from this study emphasize the importance of offering sGABO to all patients— especially those who face long wait times for gender-affirming vaginoplasty, either to complete hair removal or for other reasons, such as surgeons’ waitlist lengths, obtaining insurance or insurance approval, weight-loss, and/or deciding what gGAS to pursue. While vGABO patients experienced a greater dose reduction, they likely did not experience these benefits until a relatively later time point than if they had undergone an earlier sGABO, due to the long wait times associated with vaginoplasty. For patients who are completing sGABO as an intermediate step toward gender-affirming vaginoplasty, the scrotal scar from sGABO is not visible after later vaginoplasty, as the scrotal skin is utilized as a full-thickness skin graft to line the neovaginal canal.

It is also important to examine the reduction in GAHT and gender dysphoria described in this study within the context of the disproportionate health disparities faced by transgender and gender diverse people, as well as the limited amount of robust research into the longitudinal effects of GAHT on this patient population.^25–28^ Decreasing or discontinuing GAHT may minimize dose-dependent short- and long-term adverse effects of GAHT. It may also have the added benefit of reducing healthcare costs for a patient population that faces additional barriers in obtaining health insurance approval and in accessing providers who provide gender-affirming care.^29^ Additionally, the self-reported reduction in gender dysphoria, depression, and anxiety after GABO found in this study is notable in a patient population that faces disproportionate rates of mental health disparities and suicidal ideation.^30–32^

Compared to vGABO, sGABO can provide earlier reduction in GAHT medications and dosages, which could help reduce the greater than two-fold higher risk of death from cardiovascular disease observed amongst transgender women compared to a comparable sample of cisgender women—a finding that has been associated with estradiol GAHT.^33^^,^^34^ The more immediate benefit that patients are likely to experience relates to the day-to-day negatives associated with GAHT: financial costs, accessibility, negative side effects such as weight gain and urinary frequency, and polypharmacy.^35^^,^^36^

The importance of offering sGABO to patients pursuing vaginoplasty is further supported by patient questionnaire results. All patients who completed sGABO as a bridge to later vaginoplasty would *not* recommend against undergoing sGABO to a friend who is facing a similar wait time for vGABO. These findings are especially relevant within the current landscape of 1-3–year average wait times for vaginoplasty, as most sGABO patients reported that undergoing GABO was of greatest value when faced with a wait time for vaginoplasty of >6-12 months.^12–15^ The variability in recommended timing between sGABO and later vaginoplasty, as well as differences in self-reported preoperative priorities, highlights the importance of including patients in the surgical decision-making process. These findings may provide patients with additional information to decide between an earlier sGABO versus waiting to complete vGABO.

### Strengths and limitations

The conclusions of this study are supported by a large sample size with a high rate of questionnaire responses and by longitudinal quantitative measurements of medications and dosages. Results from this study offer both a quantitative and qualitative perspective to the positive postoperative impact of GABO, based on empirical hormone therapy dosage data and direct patient feedback. The anonymous nature of the study questionnaire supports the reliability of the results, as patients may feel more comfortable providing earnest and honest feedback.

A limitation of the study is the limited representation of different routes of GAHT intake, with analyses of transdermal estradiol and IM estradiol cypionate lacking due to a limited number of patients taking these formulations. Future studies may benefit from obtaining adequate sample sizes across GAHT routes and formulations, as well as by assessing changes in GAHT within the context of serum hormone levels and patients’ self-reported changes in secondary sex characteristics. While there was a significant reduction in GAHT after GABO observed in this study, it is important to assess GAHT dosages on a case-by-case basis to maintain adequate mental, cardiovascular, and bone health benefits.

A limitation of the study questionnaire is its retrospective design, which introduces the possibility of recall bias that may increase with the time between survey completion and surgery. Another limitation is the use of an unvalidated questionnaire, an issue that is unfortunately common with qualitative survey-based research on transgender and gender diverse populations and arises from the paucity of validated surveys for this specific patient population. We sought to address this limitation by calculating Cronbach’s alpha to assess reliability: the preoperative expectations subscale contained 4 items (α = .64), the postoperative impact subscale contained 9 items (α = .77), and the postoperative satisfaction subscale contained 5 items (α = .85).

## Conclusions

Gender-affirming bilateral orchiectomy is associated with a statistically significant decrease in gender dysphoria, estradiol, progesterone, and spironolactone, with 100% discontinuation of spironolactone. Patients who completed an earlier, standalone orchiectomy experienced improvement in all nine quality-of-life domains measured, which related to gender dysphoria, body image, concern about gender-affirming hormone therapy, and depression/anxiety. None of the patients in our study would recommend against completing an earlier, pre-vaginoplasty orchiectomy, to another patient considering this option. The majority of these patients retrospectively reported that undergoing an earlier additional outpatient surgery for standalone gender-affirming bilateral orchiectomy is worth completing when the wait time for vaginoplasty is 6-12 months or longer. The high patient satisfaction associated with standalone orchiectomy and significant reduction in all three hormone therapies suggests that patients interested in gender-affirming vaginoplasty should be offered pre-vaginoplasty orchiectomy.

## Supplementary Material

GAHT_Survey_qfae048

## Data Availability

The datasets generated and analyzed in the current study are available from the corresponding author upon request.
