# Supplementary material for: Endocrine, gender dysphoria, and sexual function benefits of gender-affirming bilateral orchiectomy: patient outcomes and surgical technique
Source: Sex Med. 2024 Aug 29;12(4):qfae048. doi: 10.1093/sexmed/qfae048 (PMC11359165; doi:10.1093/sexmed/qfae048)
Supplement: GAHT_Survey_qfae048 [file gaht_survey_qfae048.docx]

**Endocrine, Gender Dysphoria, and Sexual Function Benefits of Gender-Affirming Bilateral Orchiectomy: Patient Outcomes and Surgical Technique**

Survey Flow

Standard: Info

Block: Demographics (9 Questions)

Standard: Pre-Orchi (3 Questions)

Standard: Post-Orchi (12 Questions)

| Page Break |  |
| --- | --- |

Start of Block: Info

Q1.1
 
 
**Post-Gender Affirming Bilateral Orchiectomy**
**Treatment Satisfaction and Quality of Life**
**Study**
 
**XIRB Research Study**
**Approval #00001611**


 This questionnaire was created by a research team at 
X.

The X Institutional Review Board (IRB) Committee has reviewed and approved this study (IRB #00001611). This study is conducted in accordance with all relevant guidelines and procedures of X.
 
 
Completion and submission of this questionnaire indicates your agreement to participate in this study. This study is completely anonymous. Therefore, once the questionnaire is submitted, we will not know which responses belong to you (or any individual person). This questionnaire uses branching logic, which means that answering a question a certain way will make other questions appear or disappear. Therefore, it is essential that you answer every question. 
 
**The questionnaire should take approximately 5 minutes to complete.**
 
A focus for our research is to better understand the factors that contribute to patients' decision to undergo orchiectomy (with or without plans for future vaginoplasty) and the resultant satisfaction they have experienced post-orchiectomy.
 
In order for this work to have scientific value, it is very important that you answer each of these anonymous questions truthfully, and that you answer all of the questions provided. We greatly appreciate your contribution to this research. 
 
It is important to note that your participation is completely **anonymous**and **voluntary**. No information that could be used to identify you is collected in the questionnaire. The electronic platform used here (Qualtrics) is designed to be 100% anonymous. 
  
 Disclaimer: This questionnaire contains terms like "vaginoplasty," "orchiectomy," and "tucking." If you are uncomfortable with any part of the questionnaire, you may skip the question(s) or terminate your participation at any time.

End of Block: Info

Start of Block: Demographics

Age1 What is your age?

- ≤ 18 years old (1)
- 19-29 years old (2)
- 30-39 years old (3)
- 40-49 years old (4)
- 50-59 years old (5)
- 60-69 years old (6)
- ≥ 70 years old (7)

GAS1 Which of the following gender-affirming surgical procedures have you undergone?
**Select all that apply**

- Breast augmentation (1)
- Bilateral orchiectomy (2)
- Vaginoplasty (3)
- Facial reconstruction (4)
- Vocal modification (5)
- ⊗None of the above (6)

Skip To: End of Survey If GAS1 = None of the above

Display This Question:

If GAS1 = Vaginoplasty

GAS2 When did you undergo orchiectomy surgery?

- BEFORE my vaginoplasty surgery (1)
- At the SAME TIME of my vaginoplasty surgery (2)

| Page Break |  |
| --- | --- |

Display This Question:

If GAS2 = BEFORE my vaginoplasty surgery

GAS3 Why did you undergo orchiectomy BEFORE your vaginoplasty surgery?

- When I underwent orchiectomy, I was not planning to undergo vaginoplasty. But, after my orchiectomy, I decided to proceed with vaginoplasty surgery. (1)
- I decided to undergo orchiectomy and vaginoplasty together, BUT was offered the option to undergo "pre-vaginoplasty" orchiectomy while I waited for my vaginoplasty surgery (2)

Display This Question:

If GAS2 != At the SAME TIME of my vaginoplasty surgery

GAS4 How long ago was your **bilateral orchiectomy surgery**?

- Less than 1 month ago (1)
- Between 1 to 3 months ago (2)
- Between 3 to 6 months ago (3)
- Between 6 months to 1 year ago (4)
- More than 1 year ago (5)

Display This Question:

If GAS1 = Vaginoplasty

GAS5 How long ago was your **vaginoplasty surgery**?

- Less than 1 year ago (1)
- 1 to 2 years ago (2)
- 2 to 3 years ago (3)
- 3 to 4 years ago (4)
- 4 to 5 years ago (5)
- 5 to 10 years ago (6)
- More than 10 years ago (7)

Display This Question:

If GAS2 = BEFORE my vaginoplasty surgery

GAS6 How many **months** AFTER your orchiectomy surgery did you undergo vaginoplasty surgery?

________________________________________________________________

Display This Question:

If GAS1 != Vaginoplasty

GAS7 Is orchiectomy the only genital surgery you plan to undergo?

- Yes (1)
- I'm not sure. I may or may not undergo vaginoplasty or another genital surgery in the future (4)
- No - I PLAN TO undergo vaginoplasty or another genital surgery in the future (5)

Display This Question:

If GAS1 != Vaginoplasty

GAS8 Do you plan to undergo vaginoplasty surgery (with or without creation of a vaginal canal) in the future?

- Definitely Yes (1)
- Probably yes (2)
- Unsure (3)
- Probably not (4)
- Definitely not (5)

End of Block: Demographics

Start of Block: Pre-Orchi

Display This Question:

If GAS2 != BEFORE my vaginoplasty surgery

Q3.1 The following questions will ask about your experiences and expectations BEFORE your bilateral orchiectomy surgery.

Display This Question:

If Q3.1 Not Displayed

And GAS2 != At the SAME TIME of my vaginoplasty surgery

Q3.2 The following questions will ask about your experiences and expectations BEFORE your "pre-vaginoplasty" orchiectomy surgery.

| Page Break |  |
| --- | --- |

Display This Question:

If GAS2 != At the SAME TIME of my vaginoplasty surgery

PRE1 Rank the following potential benefits from *orchiectomy alone* or *pre-vaginoplasty orchiectomy*, in order of most to least important to you as you decided whether to proceed with bilateral orchiectomy:
 
*Please rank each statement by clicking and dragging it to order the options from most important (#1) to least important (#5).*

______ Being able to stop having to take an anti-androgen (e.g., Spironolactone) (1)

______ Being able to reduce my feminizing hormone dosage (e.g., Estrogen) (2)

______ Elimination of the majority of Testosterone from my body (3)

______ Less discomfort from having to "tuck" with testicles still present (4)

______ Looking and feeling less "masculine" from the presence of my testicles (5)

PRE2 Please rate the level of negative impact that each of the following may have had on your life BEFORE your orchiectomy:

|  | No impact (1) | Slight (2) | Moderate (3) | Significant (4) | Severe (5) |
| --- | --- | --- | --- | --- | --- |
| Gender dysphoria related to my testicles (1) |  |  |  |  |  |
| Discomfort from "tucking" (2) |  |  |  |  |  |
| Concern about the health effects of hormone therapy (3) |  |  |  |  |  |
| Side effects from Spironolactone (e.g., frequent urination) (4) |  |  |  |  |  |
| Significant masculinizing effects from Testosterone (5) |  |  |  |  |  |

Display This Question:

If GAS2 != At the SAME TIME of my vaginoplasty surgery

| 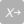 |
| --- |

PRE3 Please rate the importance of the following expectations you may have had as you considered to undergo bilateral orchiectomy:

|  | Not important (1) | Less important (2) | Neutral (3) | Important (4) | Very important (5) |
| --- | --- | --- | --- | --- | --- |
| Expected short recovery time after surgery (1) |  |  |  |  |  |
| Expected low post-operative pain (2) |  |  |  |  |  |
| Expected low rate of surgical/wound complications (3) |  |  |  |  |  |
| It affords lower hormone therapy dosage and/or number of medications following surgery (4) |  |  |  |  |  |

End of Block: Pre-Orchi

Start of Block: Post-Orchi

Q4.1 The following questions will ask about your experiences and expectations AFTER your bilateral orchiectomy surgery.

POST1 Please rate the level of any positive or negative changes that you experienced in each of the following areas AFTER your orchiectomy surgery:

|  | Not applicable to me (0) | **Was significantly worse** after orchiectomy (1) | **Was slightly worse** after orchiectomy (2) | **No change** after orchiectomy (3) | **Was slightly improved** after orchiectomy (4) | **Was significantly improved** after orchiectomy (5) |
| --- | --- | --- | --- | --- | --- | --- |
| Gender dysphoria related to my testicles (1) |  |  |  |  |  |  |
| Discomfort from "tucking" (2) |  |  |  |  |  |  |
| Concern about the health effects of hormone therapy (3) |  |  |  |  |  |  |
| Side effects from Spironolactone (e.g., frequent urination) (4) |  |  |  |  |  |  |
| Significant masculinizing effects from Testosterone (5) |  |  |  |  |  |  |
| My day-to-day general depression (6) |  |  |  |  |  |  |
| My day-to-day general anxiety (7) |  |  |  |  |  |  |
| How I feel about my body when I look at it in the mirror without clothes (8) |  |  |  |  |  |  |
| My anxiety about being seen without clothes by other people (9) |  |  |  |  |  |  |
| My comfort with being naked with a sexual partner (10) |  |  |  |  |  |  |

| Page Break |  |
| --- | --- |

Display This Question:

If GAS2 != At the SAME TIME of my vaginoplasty surgery

PAIN1 On a scale of 1-10 (with 1 = no pain, and 10 = the worst pain you have ever experienced), what was the **worst** level of pain you experienced during the first six weeks of your recovery AFTER your bilateral orchiectomy surgery?

- 1/10 (no pain) (1)
- 2/10 (2)
- 3/10 (3)
- 4/10 (4)
- 5/10 (5)
- 6/10 (6)
- 7/10 (7)
- 8/10 (8)
- 9/10 (9)
- 10/10 (worst pain I've experienced) (10)

Display This Question:

If GAS2 != At the SAME TIME of my vaginoplasty surgery

PAIN2 On a scale of 1-10 (with 1 = no pain, and 10 = the worst pain you have ever experienced), what was the **average** overall level of pain during the first six weeks of your recovery AFTER your bilateral orchiectomy surgery?

- 1/10 (no pain) (1)
- 2/10 (2)
- 3/10 (3)
- 4/10 (4)
- 5/10 (5)
- 6/10 (6)
- 7/10 (7)
- 8/10 (8)
- 9/10 (9)
- 10/10 (worst pain I've experienced) (10)

Display This Question:

If GAS2 != At the SAME TIME of my vaginoplasty surgery

PAIN3 Please rate *how qui*ckly you feel that you recovered after your bilateral orchiectomy surgery:

- Slower than I expected (1)
- About as quickly as I expected (2)
- Faster than I expected (3)

| Page Break |  |
| --- | --- |

COMP1 Did you have any post-operative complications related to your bilateral orchiectomy surgery?

- Yes (1)
- No (2)

Display This Question:

If COMP1 = Yes

COMP2 Please specify below which complications you experienced:

________________________________________________________________

Display This Question:

If COMP1 = Yes

COMP3 Did your complication(s) require surgical intervention?

- Yes (1)
- No (2)

Display This Question:

If COMP3 = Yes

COMP4 Please specify below what specific surgical intervention(s) you underwent for your complication(s):

________________________________________________________________

| Page Break |  |
| --- | --- |

Display This Question:

If GAS2 != At the SAME TIME of my vaginoplasty surgery

| 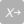 |
| --- |

SAT1 With your bilateral orchiectomy surgery, how satisfied were you with each of the following factors:

|  | Very unsatisfied (1) | Unsatisfied (2) | Neutral (3) | Satisfied (4) | Very satisfied (5) |
| --- | --- | --- | --- | --- | --- |
| Rate of recovery (time required to complete recovery) (1) |  |  |  |  |  |
| Tolerability of post-operative pain (2) |  |  |  |  |  |
| Ease of care of the surgical wound (3) |  |  |  |  |  |
| Appearance of the scar ≥6 weeks after surgery (4) |  |  |  |  |  |
| Effect of the surgery to make the genitals look "less male" (5) |  |  |  |  |  |

Display This Question:

If GAS2 = BEFORE my vaginoplasty surgery

SAT2 Would you recommend a pre-vaginoplasty orchiectomy to a friend who is considering vaginoplasty surgery?

- Yes (1)
- Maybe (2)
- No *(please specify why)*: (3) __________________________________________________

Display This Question:

If GAS2 = BEFORE my vaginoplasty surgery

SAT3
As someone who underwent vaginoplasty sometime AFTER their orchiectomy, was it "worth it" to you to undergo orchiectomy as a separate surgery before vaginoplasty?

- Yes (1)
- No (2)

Display This Question:

If SAT3 = Yes

SAT4
Up to how many months BEFORE vaginoplasty surgery would you say it is still "worth it" to undergo pre-vaginoplasty bilateral orchiectomy?

Months (1)

▼ 1 month (1) ... Greater than 12 months (13)

End of Block: Post-Orchi
